# Scalable Green Approach Toward Fragrant Acetates

**DOI:** 10.3390/molecules25143217

**Published:** 2020-07-14

**Authors:** Eva Puchl’ová, Peter Szolcsányi

**Affiliations:** Department of Organic Chemistry, Slovak University of Technology, Radlinského 9, 81237 Bratislava, Slovakia; eva.puchlova@stuba.sk

**Keywords:** enzymatic acetylation, green solvents, chemo-selectivity

## Abstract

The advantageous properties of ethylene glycol diacetate (EGDA) qualify it as a useful substitute for glycerol triacetate (GTA) for various green applications. We scrutinised the lipase-mediated acetylation of structurally diverse alcohols in neat EGDA furnishing the range of naturally occurring fragrant acetates. We found that such enzymatic system exhibits high reactivity and selectivity towards activated (homo) allylic and non-activated primary/secondary alcohols. This feature was utilised in the scalable multigram synthesis of fragrant (*Z*)-hex-3-en-1-yl acetate in 70% yield. In addition, the Lipozyme 435/EGDA system was also found to be applicable for the chemo-selective acetylation of (hydroxyalkyl) phenols as well as for the kinetic resolution of chiral secondary alcohols. Lastly, its discrimination power was demonstrated in competitive experiments of equimolar mixtures of two isomeric alcohols. This enabled the practical synthesis of 1-pentyl acetate isolated as a single product in 68% yield from the equimolar mixture of 1-pentanol and 3-pentanol.

## 1. Introduction

In perfumery and the flavour industry, acetates are the most important aliphatic esters used and valued for their typical aromas [1]. Although most of them are natural products, their isolation from sources of origin makes the process economically unfeasible. Therefore, numerous chemical approaches toward fragrant acetates were successfully developed using homogenous or heterogeneous acid, base, or transition-metal catalysis [2]. However, perfumery and flavour grade esters need to be free of inorganic impurities and undesirable colouration. Moreover, increasing the health consciousness and environmental issues strongly encourages the development of benign yet efficient biocatalytic protocols as viable green alternatives [3]. Among them, the use of lipases gained considerable attention due to their high reactivity and remarkable selectivity under diverse reaction conditions [4]. In addition, their stability in a non-aqueous environment, particularly under solvent-free conditions, gives lipases a comparative advantage over other biocatalysts [5]. In this context, glycerol-based solvents [6,7,8] and/or reagents represent a green alternative to commonly used reaction media. One of the perspective glycerol’s derivatives is triacetin (glycerol triacetate, GTA), which was recently used both as a reagent and solvent for the enzymatic acetylation of some alcohols [9,10,11,12]. A similar and commercially available derivative, ethylene glycol diacetate (EGDA) can be considered an interesting alternative to GTA for analogous transformations. With the lower boiling point and viscosity, better recyclability, and regeneration, EGDA has a promising synthetic potential for the scalable preparation of natural acetates for perfumery and/or flavour purposes.

Therefore, we have decided to scrutinise the practical use of EGDA for the enzymatic acetylation of selected alcohols that would afford known and naturally occurring fragrant acetates. In addition, we were interested in the chemo-selectivity of such a reaction system with respect to the structure and/or reactivity of substrates of commercial interest.

## 2. Results and Discussion

**Scheme 1:**
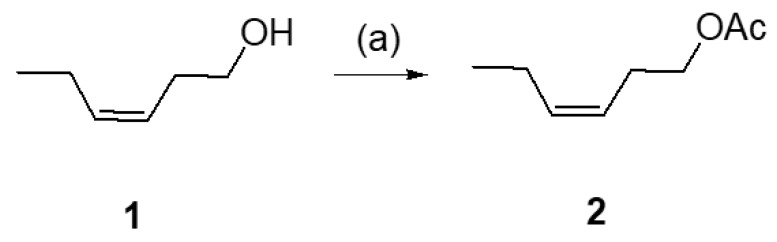
Optimised preparation of fragrant *(Z*)-hex-3-ene-1-ol **1**. Reagents and conditions: (**a**) Ethylene glycol diacetate (EGDA) (2 equiv), Lipozyme 435 (2% wt), 40 °C, 150 rpm, 70% yield (20 g scale).

The initial reaction screening was done on (*Z*)-hex-3-ene-1-ol **1** as a model substrate, which was acetylated [13,14,15] either in neat EGDA or with the cosolvent (MTBE or hexane) using Lipozyme 435 as a biocatalyst to afford (*Z*)-hex-3-ene-1-yl acetate **2**. The enzyme and EGDA loadings as well as temperature, concentration, and co-solvent were scrutinised as variable parameters while the progress of the reaction was monitored by GC-FID (see Table S1 in the Supporting Information). The identified optimal reaction conditions were then applied for the scale-up preparation of fragrant acetate **2** in 70% yield. It is noteworthy that the isolation and purification protocol involves environmentally benign procedures only (filtration, hydrodistillation, and vacuum rectification) and is free of chromatography and/or any additional organic solvents (Scheme 1).

**Scheme 2:**
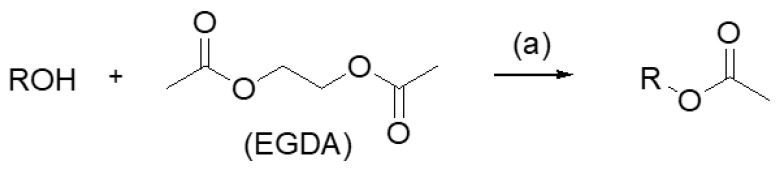
Reaction screening of the enzymatic acetylation. Reagents and conditions: (**a**) EGDA (2 equiv), Lipozyme 435 (2% wt), 40 °C, 150 rpm, Table 1.

Next, we have applied the optimal reaction conditions in the acetylation screening of various alcohols (Scheme 2) that furnish naturally occurring fragrant acetates (entries 1–14, Table 1). In parallel, we have investigated the chemo-selectivity of such an enzymatic system for discrimination between structurally different alcohols by adding more substrates (entries 15–20, Table 1)—regardless of aroma properties of their acetates—to the screening for comparison purposes. The obtained results clearly show the following reactivity patterns.

(a) Considering the activated substrates, allylic primary alcohols **3**, **5**, **7**, **9**, **13**, and **15** (entries 2–5, 7, 8) were almost quantitatively esterified (GC-FID ratios of alcohol to acetate ranging from 10:90 to 4:96) to fragrant acetates, namely geranyl acetate **4** [16,17,18,19,20,21,22,23,24,25,26,27,28,29,30,31,32,33,34], prenyl acetate **6**, (*E*)-2-hexenyl acetate **8**, cinnamyl acetate **10** [35,36,37,38,39,40,41], phytyl acetate (*E*/*Z* = 66:34) 14 [42], and (1*R*)-nopyl acetate **16**, respectively. On the other hand, *rac*-linalool **11** remained almost intact with negligible acetylation only (entry 6). Furthermore, the enzyme clearly discriminates between the primary and tertiary allylic alcohol. Analogously, the primary homoallylic alcohols **1** and **17** also provided corresponding fragrant acetates (entries 1 and 9), namely (*Z*)-hex-3-ene-1-yl acetate **2** and (1*R*)-myrtenyl acetate **18**.
(b) Regarding the non-activated substrates, the enzyme clearly distinguishes primary alcohols **27**, **29** (entries 15, 16), and/or secondary alcohols **19**, **21**, **23**, **33**, and **35** (entries 10–13, 18, 19) vs. phenolic eugenol **31** (entry 17) and/or tertiary alcohol **37** (entry 20). While the former ones were reasonably acetylated (although not as well as activated alcohols, vide supra) to their corresponding acetates **20**, **22**, **24**, **28**, **30**, **34**, and **36**, the latter ones were fully reluctant to the transformation. In addition, the enzyme´s enantio-discrimination led to the kinetic resolution of racemic secondary alcohols **19**, **21** (entries 10, 12). In the case of *rac*-2-heptanol **19** (entry 10), (*R*)-enantiomer was mostly acetylated [43,44,45,46,47,48,49] as shown by negligible formation of (*S*)-2-heptenyl acetate (*S*)-**20** (see Figure S1 in the Supporting Information), and, thus, by obeying the Kazlauskas’s rule [50] (entry 11). Conversely, (1*R*)-*endo*-fenchol **25** remained practically intact. This might be due to the significant steric congestion around the hydroxyl group and/or the result of the enantiomer preference of the enzyme for the opposite enantiomer (entry 14). In addition, the enzymatic acetylation [51,52,53] of bifunctional *rac*-rhododendrol **21** exhibited both the chemo-selectivity (alcohol vs. phenol) and the kinetic resolution of racemate (entry 12). Thus, the secondary hydroxyl group of the (*R*)-enantiomer (cf. the Kazlauskas’s rule again) was mostly acetylated to furnish the ester (*R*)-**22** along with the enantiomerically enriched (*S*)-rhododendrol (*S*)-**21** (84% *ee*, chiral HPLC, see Figure S2 in the Supporting Information). The clear unreactivity of phenols under the reaction conditions was also exhibited by zero acetylation of eugenol **31** (entry 17).
(c) Considering the achiral secondary alcohols, even minor structural differences led to a significant change in their reactivity. While isopropanol **35** (entry 19) was acetylated in a major extent (GC-FID ratio **35**/**36** = 15:85), slightly larger pentan-3-ol **33** (entry 18) was much less reactive (GC-FID ratio **33**/**34** = 45:55). The extent of acetylation of 2-indanol **23** (entry 13), being somewhere in between (GC-FID ratio **23**/**24** = 24:76), might indicate the high sensitivity of the respective lipase to the steric demands of the alcohols in question.

**Scheme 3:**
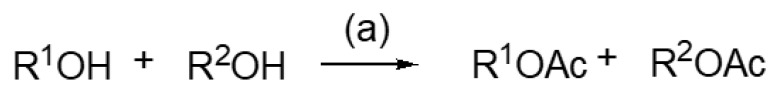
Chemo-selective acetylation of equimolar mixtures. Reagents and conditions: (**a**) alcohols (1:1), ethylene glycol diacetate (EGDA) (1 equiv), Lipozyme 435 (1% wt), 40 °C, 150 rpm, Table 2.

**Figure 1:**
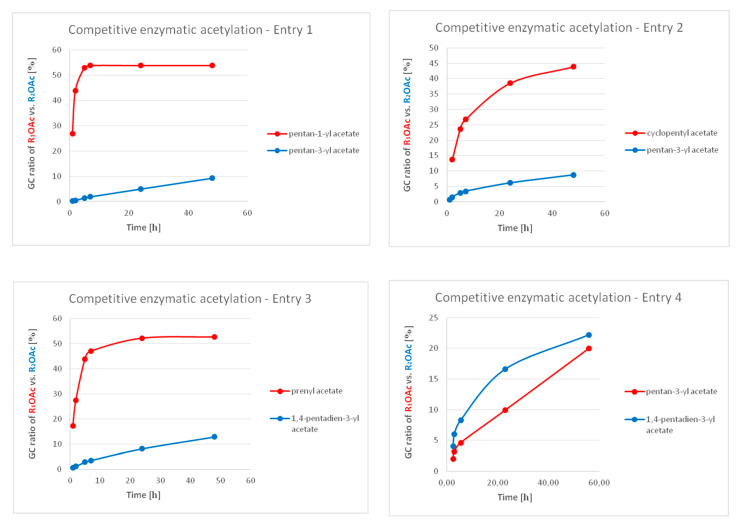
Time-dependent evolution of the ratio between forming acetates during competitive experiments (cf. Scheme 3, cf. Tables S3–S6 in the Supporting Information).

Next, we aimed to practically utilise the observed chemo-selectivity of the enzymatic system, and, therefore, we tested its discrimination power on two-component mixtures of similar yet different alcohols. Thus, we have performed competitive acetylation experiments with equimolar mixtures of four pairs of alcohols: (a) 1-pentanol **39** vs. 3-pentanol **33**, (b) 3-pentanol **33** vs. cyclopentanol **41**, (c) prenol **5** vs. divinylcarbinol **43**, and (d) 3-pentanol **33** vs. divinylcarbinol **43** (Scheme 3 and Table 2). The obtained results clearly show that lipase-catalysed acetylation provided excellent discrimination between primary and secondary alcohols in favour of the former ones (Table 2, entries 1 and 3). Thus, primary 1-pentanol **39** was almost exclusively acetylated to 1-pentyl acetate **40** (GC-FID ratio **39**/**40** = 6:94) at the expense of its secondary isomer 3-pentanol **33** that barely afforded its acetate **34** (GC-FID ratio **33**/**34** = 96.5:3.5). Similarly, primary allylic prenol **5** was overwhelmingly acetylated to prenyl acetate **6** (GC-FID ratio **5**/**6** = 9:91) in the presence of secondary divinylcarbinol **43** that was concomitantly transformed to its acetate **44** in a minor extent only (GC-FID ratio **43**/**44** = 81:19). On the other hand, the chemo-selectivity of competitive enzymatic acetylation of cyclic vs. acyclic secondary alcohols was less pronounced (Table 2, entry 2). Although cyclopentanol **41** was acetylated much faster to its acetate **42** (GC-FID ratio **41**/**42** = 19:81) than acyclic 3-pentanol **33** to 3-pentyl acetate **34** (GC-FID ratio **33**/**34** = 80:20) due to the necessary longer reaction time, the discrimination factor was inevitably reduced. On the other hand, the preferential acetylation of **41** over **33** might be attributed to the higher reactivity of less sterically hindered and more nucleophilic hydroxyl group of cyclopentanol **41** when compared to conformationally more flexible 3-pentanol **33**. Lastly, the reactivity of non-activated (alkylic) vs. activated (allylic) secondary alcohols was comparable. This was evident from the competitive acetylation of an equimolar mixture of 3-pentanol **33** and divinylcarbinol **43**, which generated their corresponding acetates **34** and **44** without significant discrimination between each other (Table 2, entry 4). The time-dependent evolution of the ratio between forming acetates is depicted in Figure 1. For the corresponding analytical data, see Tables S3–S6 in the Supporting Information.

**Scheme 4:**
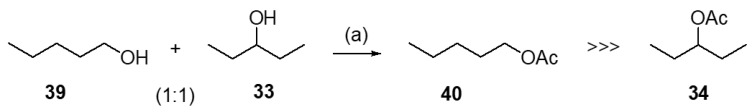
Chemo-selective acetylation of equimolar mixture of alcohols. Reagents and conditions: (**a**) **39** + **33** (1:1), EGDA (1 equiv), Lipozyme 435 (1% wt), 40 °C, 150 rpm, 4.5 h, 40 (94% GC-FID purity, 68% yield from **39**).

The observed enzymatic discrimination between interrelated alcohols could be potentially used for the chemo-selective acetylation of their mixtures and/or for the reactive separation of product-of-choice from a multi-component (raw) material. To verify such an approach on a model system, the preparative-scale enzymatic acetylation of an equimolar mixture (5 g each) of 1-pentanol **39** and 3-pentanol **33** was carried out. In order to maximise the chemo-selectivity, we have lowered the amount of both the enzyme and EGDA loading by half in comparison to standard conditions (cf. Scheme 2). Thus, after gentle heating and stirring the reaction mixture for less than 5 h, which was followed by its hydrodistillation and final extraction of distillate, 1-pentyl acetate **40** was isolated in 68% yield (Scheme 4).

## 3. Materials and Methods

### 3.1. Materials and Methods

Chemicals and reagents were purchased from commercial sources (Alfa Aesar, Sigma-Aldrich, Merck) and were used without further purification. Hexanes refer to a mixture of C-6 alkanes (b.p. 60–80 °C). Lipozyme 435 (*Candida antarctica lipase B* immobilized on a macroporous acrylic resin) with a reported activity of 8000 PLU (propyl laurate units) per gram was purchased from Novozymes A/S (Bagsværd, Denmark). All GC-FID analyses were performed on a gas chromatograph Agilent 7890A equipped with FID and a split–splitless injector as follows: (a) standard GC-FID using column DB-WAX (30 m × 0.25 mm × 0.15 µm), for rhododendrol **21**: injection 1.0 µL, split 20:1, temperature gradient 60 °C (0 min) → 20 °C/min → 200 °C (25 min), carrier gas H_2_, flow 1.2 mL/min, for all the rest of alcohols (except phytol **13**): injection 0.01 µL, split 50:1, temperature gradient 40 °C (0 min) → 10 °C/min → 200 °C (12 min), carrier gas H_2_, flow 1.2 mL/min, (b) standard GC-FID for phytol **13** using column DB-5 (30 m × 0.25 mm × 0.25 µm), injection 0.01 µL, split 50:1, temperature gradient 40 °C (0 min) → 10 °C/min → 200 °C (12 min), carrier gas H_2_, flow 1.2 mL/min, (c) chiral GC-FID using column Lipodex-A (50 m × 0.25 mm × 0.25 µm), injection 0.2 µL, split 50:1, 55 °C (50 min) isothermal, carrier gas H_2_, flow 2.0 mL/min). All GC-MS analyses (except rhododendrol **21**) were performed on a gas chromatograph Agilent 7890A coupled with Agilent 5975C inert MSD with Triple-Axis Detector (column DB-Wax 30 m × 0.25 mm × 0.15 µm, injection 0.1 µL, split 20:1, temperature gradient 40 °C (0 min) → 10 °C/min → 200 °C (12 min), carrier gas H_2_, flow 1.0 mL/min), for rhododendrol **21**: temperature gradient 60 °C (0 min) → 20 °C/min → 220 °C (25 min), carrier gas H_2_, flow 1.0 mL/min). To monitor the reaction progress, the relative amount (reported as a percentage) of each component (alcohol vs. acetate) was determined according to the proportion of each peak area in the total peak area of the two compositions. The chemical identity of prepared acetates was determined as follows: (a) GC-FID comparison with the commercial standard for **2**, **4**, **6**, **8**, *rac*-**20**, (*S*)-**20**, (b) GC-MS (NIST library) for **10**, **12**, **14**, **16**, **18**, *rac*-**20**, (*S*)-**20**, *rac*-**22**, **24**, **26**, **28**, **34**, **36**, (c) ^1^H NMR for *rac*-**22**, **30**. NMR spectra were recorded on a Varian INOVA 300 spectrometer and were correctly shifted using residual non-deuterated solvent (CHCl_3_: δ_H_ = 7.26 ppm). Chiral HPLC analyses were performed on Agilent 1260 Infinity LC system equipped with a Lux^®^ Amylose-1 column (250 × 4.6 mm, 5 µm, stationary phase: amylose *tris*-(3,5-dimethylphenyl) carbamate, Phenomenex, Torrance, CA, USA) and diode-array detector (220 nm). The separation was carried out at 15 °C by isocratic elution with *n*-hexane/isopropanol (90:10, *v*/*v*) with the flow rate of 1 mL/min.

### 3.2. Synthetic Procedures and Analytical Data

General experiment for the optimisation screening of acetylation of (Z)-hex-3-en-1-ol (**1**):

To a solution of an alcohol (10.0 mmol) in either neat EGDA or EGDA/cosolvent mixture was added Lipozyme 435. The reaction mixture was stirred in an orbital shaker (150 rpm) at two different temperatures (40 or 45 °C, respectively) and the reaction progress was monitored by GC-FID (see Table S1 in the Supporting Information).

General experiment for the optimised acetylation of alcohols, according to Scheme 2:

Lipozyme 435 (2% wt) was added to a solution of an alcohol (1 equiv) in EGDA (2 equiv). The reaction mixture was incubated in an orbital shaker (40 °C, 150 rpm) and the reaction progress was monitored by GC-FID (see Table 1).

Scale-up preparation of (Z)-hex-3-en-1-yl acetate (**2**):

Lipozyme 435 (400 mg, 2% wt) was added to a solution of (*Z*)-hex-3-en-1-ol **1** (20.0 g, 0.2 mol) in EGDA (58.3 g, 0.4 mol). The reaction mixture was incubated in an orbital shaker (40 °C, 150 rpm) and the reaction progress was monitored by GC-FID. The enzyme was filtered off and the filtrate was hydro-distilled. The separated organic phase was purified by vacuum rectification (b.p. 62–63 °C/20 mbar) affording (*Z*)-hex-3-en-1-yl acetate **2** in two fractions as colourless oils: first fraction (8.11 g, 80% GC-FID purity, containing 6.48 g of **2**) and second fraction (13.35 g, 95.7% GC-FID purity, containing 12.80 g of **2**) in a combined 70% yield. ^1^H NMR (300 MHz, CDCl_3_): δ = 5.53 − 5.45 (m, 1H, H-3), 5.35 − 5.26 (m, 1H, H-4), 4.05 (t, *J* = 6.9 Hz, 2H, H-1), 2.39 − 2.32 (m, 2H, H-2), 2.09 − 2.00 (m, 5H, H-5, Me), 0.96 (t, *J* = 7.5 Hz, 3H, H-6). ^13^C NMR (75 MHz, CDCl_3_): δ = 171.3 (C = O), 134.7 (C-4), 123.8 (C-3), 64.1 (C-1), 26.8 (C-2), 21.1 (Me), 20.7 (C-5), 14.3 (C-6). The obtained data fully correspond to the reported spectra [54].

Enzymatic acetylation of rhododendrol (**21**) for the identification purposes:

Lipozyme 435 (10 mg, 2% wt) was added to a suspension of racemic rhododendrol **21** (0.50 g, 30.1 mmol) in EGDA (0.88 g, 60.2 mmol, 2 equiv). The reaction mixture was incubated in an orbital shaker (40 °C, 150 rpm) and GC-FID monitored the progress of the reaction. The enzyme was filtered off, the filtrate was diluted with Et_2_O (10 mL), and it was washed with 2M aq. NaOH (15 mL) to separate the unreacted enantiomer of the substrate [55]. The aqueous layer was washed with Et_2_O (10 mL) and acidified with 9% aq. HCl to pH 6. After extraction of the aqueous phase with Et_2_O (10 mL), the separated organic phase was dried over anhydrous Na_2_SO_4_, filtered, and evaporated in vacuo to furnish an oil. An aliquot sample of the crude material was subjected to proton NMR [56] and GC-MS [57] analyses to identify the acetate **22**, and the obtained spectra fully corresponded to the reported data. In the alternative experiment, after completion of the reaction, the enzyme was filtered off, the filtrate was diluted with water, and it was extracted with methyl tert-butyl ether (MTBE). The separated organic phase was dried over Na_2_SO_4_ and concentrated *in vacuo* to furnish an oil. An aliquot sample of the crude material was purified by flash liquid chromatography (FLC) (silica gel, DCM/MeOH 95:5) to obtain two fractions: the first one containing the less polar acetate **22** (R_f_ = 0.60) and the second fraction containing the more polar alcohol **21** (R_f_ = 0.34). The latter one was then analyzed on chiral HPLC and was found to contain enantiomerically enriched (*S*)-rhododendrol (*S*)-**21** (*e.r.* = 92/8, see Figure S2 in the Supporting Information, the enantiomeric elution order was adopted from Reference [58]).

Enzymatic acetylation of pent-3-yn-1-ol (**29**) for identification purposes:

Lipozyme 435 (20 mg, 2% wt) was added to a solution of pent-3-yn-1-ol **29** (1.0 g, 11.9 mmol) in EGDA (3.47 g, 23.8 mmol, 2 equiv). The reaction mixture was incubated in an orbital shaker (40 °C, 150 rpm) and the reaction progress was monitored by GC-FID. The enzyme was filtered off and the filtrate was extracted with Et_2_O (3 × 15 mL). The separated organic layer was dried over anhydrous Na_2_SO_4_, filtered, and evaporated in vacuo. An aliquot of the crude material was purified by FLC on silica gel (hexanes/AcOEt 1:1) to obtain an analytical sample of pure acetate **30**. ^1^H NMR (300 MHz, CDCl_3_): δ = 4.12 (t, *J* = 6.9 Hz, 1H, H-1), 2.46 (ddt, *J* = 6.9, 4.4, 2.6 Hz, 1H, H-2), 2.07 (s, 1H, Me), 1.78 (t, *J* = 2.6 Hz, 1H, H-5).

General experiment for the competitive acetylations, according to Scheme 3.

EGDA (1 equiv) and Lipozyme 435 (1% wt) was added to an equimolar mixture of two alcohols (0.5 + 0.5 equiv). The reaction mixture was stirred in an orbital shaker (40 °C, 150 rpm) and the reaction progress was monitored by GC-FID. (see Table 2).

Chemo-selective acetylation of pentan-1-ol (**39**):

EGDA (16.6 g, 113.4 mmol) and Lipozyme 435 (100 mg, 1% wt) was added to an equimolar mixture of pentan-1-ol **39** (5.0 g, 56.7 mmol) and pentan-3-ol **33** (5.0 g, 56.7 mmol). The reaction mixture was stirred in an orbital shaker (40 °C, 150 rpm) and the reaction progress was monitored by GC-FID. The enzyme was filtered off and the filtrate was hydro-distilled. The separated organic phase was diluted with hexane (75 mL), and washed with aq. NaHCO_3_ soln. (3 × 75 mL, pH 9) and distilled water (150 mL). The separated organic phase was dried over Na_2_SO_4_ and concentrated *in vacuo* (40 °C, 500 → 200 mbar) to yield pent-1-yl acetate **40** as colourless oil (5.3 g, 94% GC-FID purity, 68% yield from **39**). ^1^H NMR (300 MHz, CDCl_3_): δ = 4.03 (t, *J* = 6.8 Hz, 2H, H-1), 2.02 (s, 3H, Me), 1.64 − 1.56 (m, 2H, H-2), 1.31 (m, 4H, H-3, H-4), 0.90 − 0.87 (m, 3H, H-5). ^13^C NMR (75 MHz, CDCl_3_): δ = 171.3 (C = O), 64.7 (C-1), 28.4 (C-2), 28.2 (C-3), 22.4 (C-4), 21.1 (Me), 14.0 (C-5). The obtained data fully correspond to the reported spectra [59].

## 4. Conclusions

In combination with Lipozyme 435, we have successfully employed EGDA as a useful acetylation reagent and solvent for chemo-selective and/or stereoselective enzymatic preparation of natural acetates for perfumery and/or flavour purposes. We found that such a green system exhibits high reactivity and selectivity toward activated (homo) allylic and non-activated primary/secondary alcohols. This feature was utilised in the scalable multigram synthesis of fragrant (*Z*)-hex-3-en-1-yl acetate **2** in 70% yield after vacuum rectification. In addition, the Lipozyme 435/EGDA system was also found to be applicable to the chemo-selective acetylation of (hydroxyalkyl) phenols as well as to the kinetic resolution of chiral secondary alcohols. Lastly, its remarkable discrimination power enabled the preferential acetylation of respective alcohol from the equimolar mixture of two isomeric substrates. Thus, pure 1-pentyl acetate **40** was isolated as a single product in 68% yield in a multigram quantity from the 1:1 mixture of 1-pentanol **39** and 3-pentanol **33**.

## Figures and Tables

**Table 1:** Screening of the enzymatic acetylation of alcohols according to Scheme 2.

Entry	Alcohol	Acetate	Time (h)	GC-FID Ratio ^a^ (%)
1	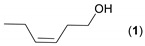	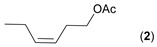	19	6:94
2	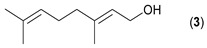	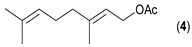	24	5:95
3	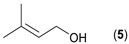	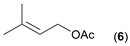	23	4:96
4	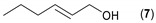	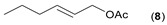	24	5:95
5	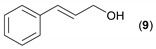	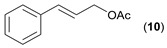	48	8:92
6	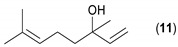	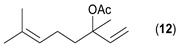	72	91:9
7	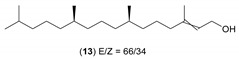	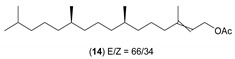	24	4:96
8	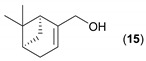	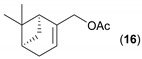	24	10:90
9	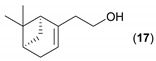	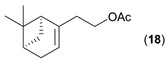	24	6:94
10	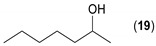	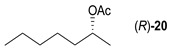	20	48:52
11	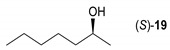	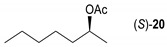	72	91:9
12	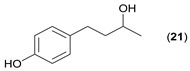	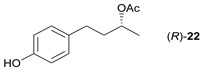	48	52:48
13	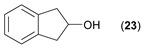	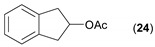	72	24:76
14	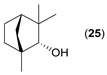	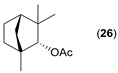	48	98:2
15	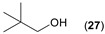	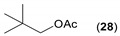	72	14:86
16	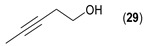	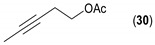	42	11:89
17	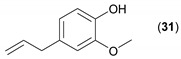	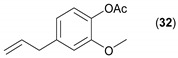	48	100:0
18	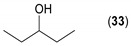	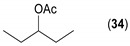	51	45:55
19	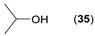	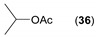	48	15:85
20	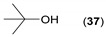	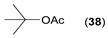	48	100:0

^a^ The compositions were obtained by GC-FID analysis by comparing peak areas of alcohol vs. acetate.

**Table 2:** Screening of competitive enzymatic acetylation of equimolar mixtures of alcohols according to Scheme 3.

Entry	Substrates R^1^OH + R^2^OH	Products R^1^OAc + R^2^OAc	Time (h)	R^1^OH/R^1^OAc (GC-FID Ratio %) ^a^	R^2^OH/R^2^OAc (GC-FID Ratio %) ^a^
1	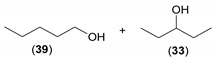	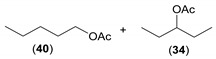	8	**39/40** (6:94)	**33/34** (96.5:3.5)
2	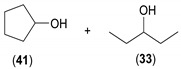	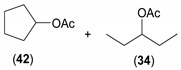	48	**41/42** (19:81)	**33/34** (80:20)
3	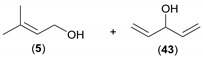	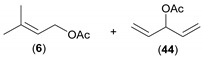	24	**5/6** (9:91)	**43/44** (81:19)
4	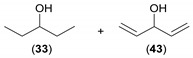	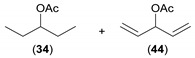	56	**33/34** (66:34)	**43/44** (51:49)

^a^ The compositions were obtained by GC-FID analysis by comparing peak areas of respective alcohol vs. its acetate.

## Supplementary Materials

The following are available online: optimisation screening conditions, GC-FID retention times of all compounds, copies of chiral GC-FID and HPLC spectra, copies of NMR spectra of isolated compounds.
