# Supplementary material for: Scalable Green Approach Toward Fragrant Acetates
Source: Molecules. 2020 Jul 14;25(14):3217. doi: 10.3390/molecules25143217 (PMC7397122; doi:10.3390/molecules25143217)
Supplement: Supplementary file 1 [file molecules-25-03217-s001.pdf]

molecules

Article

Scalable Green Approach Toward Fragrant Acetates

Eva Puchl'ová and Peter Szolcsányi *

Department of Organic Chemistry, Slovak University of Technology, Radlinského 9, 81237 Bratislava, Slovakia; eva.puchlova@stuba.sk
* Correspondence: peter.szolcsanyi@stuba.sk

Received: 18 June 2020; Accepted: 13 July 2020; Published: date

Contents

Contents	
**Optimisation screening of enzymatic acetylation of (*Z*)-hex-3-en-1-ol**	**Page 1**
**GC-FID retention times of respective alcohols and corresponding acetates**	**Pages 2-3**
**Time-dependent GC-FID ratios of competitive enzymatic acetylations**	**Pages 3-4**
**Chiral GC-FID analyses of heptan-2-ol and heptan-2-yl acetate**	**Page 5**
**Chiral HPLC analysis of rhododendrol**	**Page 6**
**Copies of NMR spectra of isolated compounds**	**Pages 6-8**

Table S1. Optimisation screening of enzymatic acetylation of (*Z*)-hex-3-en-1-ol (1).

Entry	Lipozyme 435 [% wt]	EGDA [equiv]	Temperature [°C]	Cosolvent (c) [mol. L^{-1}]	Reaction time [h]	GC-FID ratio (1)/(2)[a] [%]
1	10	4.5	40	-	5.5	3/97
2	1	4.5	40	-	18	3/97
3	1	3	40	-	22	4/96
4	1	1.5	40	-	24	9/91
5	1	1.5	45	-	17	9/91
6	2	1.5	40	-	24	9/91
7	2	2	40	-	19	6/94
8	2	1	40	-	16	15/85
9	2	0.5	40	-	16	29/71
10	1	0.5	40	MTBE (2.0)	44	32/68
11	1	1	40	MTBE (2.0)	44	15/85
12	1	1	40	MTBE (4.0)	44	14/86
13	1	1	40	Hexane (4.0)	44	12/88

a) The compositions were obtained by GC-FID analysis by comparing peak areas of alcohol **1** *vs.* acetate **2.**

Table S2. GC-FID retention times of respective alcohols and corresponding acetates.

Entry	Alcohol	Retention time [min]	Acetate	Retention time [min]
1	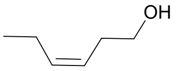 (**1**)	7.849	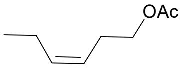 (**2**)	5.996
2	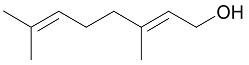 (**3**)	10.956	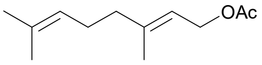 (**4**)	11.935
3	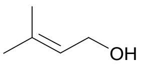 (**5**)	4.129	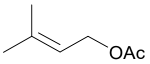 (**6**)	5.051
4	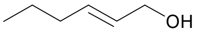 (**7**)	6.950	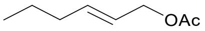 (**8**)	6.026
5	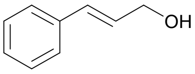 (**9**)	14.790	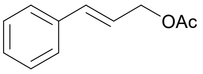 (**10**)	15.982
6	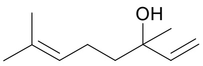 (**11**)	13.797	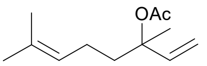 (**12**)	13.521
7	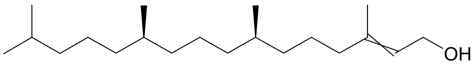 (**13**) E/Z = 66/34	21.781/22.453	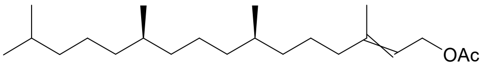 (**14**) E/Z = 66/34	24.843/26.046
8	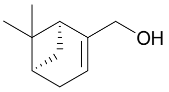 (**15**)	11.952	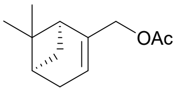 (**16**)	11.243
9	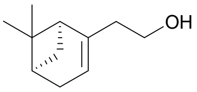 (**17**)	11.358	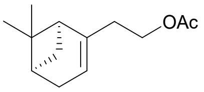 (**18**)	10.253
10	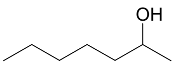 (**19**)	5.900	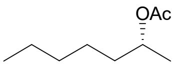 (*R*)-**20**	5.202
11	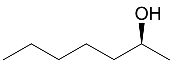 (*S*)-**19**	5.900	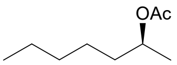 (*S*)-**20**	5.202
12[b]	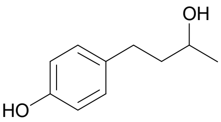 (**21**)	15.795	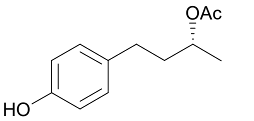 (*R*)-**22**	13.797
13	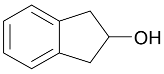 (**23**)	14.674	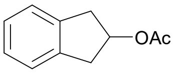 (**24**)	13.609
14	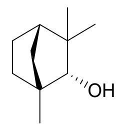 (**25**)	9.043	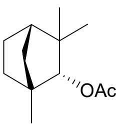 (**26**)	8.921
15	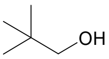 (**27**)	3.556	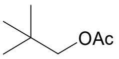 (**28**)	2.729
16	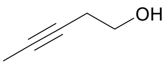 (**29**)	7.538	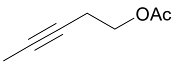 (**30**)	7.128
17	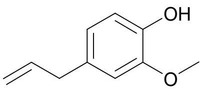 (**31**)	14.930	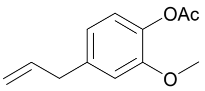 (**32**)	n.a.

18	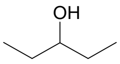 **(33)**	3.445	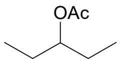 **(34)**	3.095
19	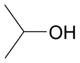 **(35)**	2.300	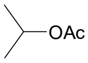 **(36)**	2.098
20	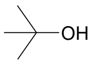 **(37)**	2.139	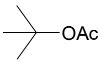 **(38)**	n.a.

Table S3. Time-dependent GC-FID ratios of competitive enzymatic acetylation of 1-pentanol (39) *vs.* 3-pentanol (33).

Reaction time [h]	Reaction temperature [°C]	Pentan-1-ol (39) t_R = 5.051 min	Pentan-1-yl acetate (40) t_R = 4.129 min	Pentan-3-ol (33) t_R = 3.445 min	Pentan-3-yl acetate (34) t_R = 3.095 min
1	28	26.8	26.8	46.3	0.1
2	40	11.6	43.8	44.2	0.4
5	40	4.0	52.8	41.9	1.3
7	40	3.4	53.8	41.0	1.8
24	40	3.4	53.8	37.9	4.9
48	40	3.4	53.8	33.6	9.2

GC-FID: column DB-Wax (30 m x 0.25 mm x 0.15 μm), injection 0.01 μL, split 50:1, temperature gradient 40 °C (0 min) → 10 °C/min → 200 °C (12 min), carrier gas H_2 (1.2 mL/min). The compositions were obtained by comparing peak areas of both alcohols and acetates.

Table S4. Time-dependent GC-FID ratios of competitive enzymatic acetylation of cyclopentanol (41) *vs.* 3-pentanol (33).

Reaction time [h]	Reaction temperature [°C]	Cyclopentanol (41) t_R = 5.667 min	Cyclopentyl acetate (42) t_R = 5.036 min	Pentan-3-ol (33) t_R = 3.445 min	Pentan-3-yl acetate (34) t_R = 3.095 min
1	28	43.6	7.7	48.0	0.7<br>0,.0
2	40	39.3	13.8	45.4	1.5
5	40	30.2	23.7	43.2	2.9
7	40	27.9	26.8	41.9	3.4
24	40	16.5	38.6	38.8	6.1
48	40	11.0	43.9	36.4	8.7

GC-FID: column DB-Wax (30 m x 0.25 mm x 0.15 μm), injection 0.01 μL, split 50:1, temperature gradient 40 °C (0 min) → 10 °C/min → 200 °C (12 min), carrier gas H_2 (1.2 mL/min). The compositions were obtained by comparing peak areas of both alcohols and acetates.

Table S5. Time-dependent GC-FID ratios of competitive enzymatic acetylation of prenol (5) *vs.* divinylcarbinol (43).

Reaction time [h]	Reaction temperature [°C]	Prenol (5) t_R = 5.911min	Prenyl acetate (6) t_R = 5.051 min	1,4-Pentadien-3-ol (43) t_R = 4.888 min	1,4-Pentadien-3-yl acetate (44) t_R = 3.871 min
1	28	40.1	17.2	42.1	0.6
2	40	28.6	27.5	42.7	1.2
5	40	12.4	43.8	41.0	2.8
7	40	9.0	47.0	40.5	3.5
24	40	5.8	52.2	33.9	8.1
48	40	5.2	52.7	29.3	12.8

GC-FID: column DB-Wax (30 m x 0.25 mm x 0.15 μm), injection 0.01 μL, split 50:1, temperature gradient 40 °C (0 min) → 10 °C/min → 200 °C (12 min), carrier gas H_2 (1.2 mL/min). The compositions were obtained by comparing peak areas of both alcohols and acetates.

Table S6. Time-dependent GC-FID ratios of competitive enzymatic acetylation of 3-pentanol (33) *vs.* divinylcarbinol (43).

Reaction time [h]	Reaction temperature [°C]	Pentan-3-ol (33) t_R = 3.445 min	Pentan-3-yl acetate (34) t_R = 3.095 min	1,4-Pentadien-3-ol (43) t_R = 4.888 min	1,4-Pentadien-3-yl acetate (44) t_R = 3.871 min
2.5	28.5	50.0	2.0	44.0	4.0
3	40	48.9	3.2	41.9	6.0
5.5	40	46.4	4.6	40.7	8.3
23	40	39.9	9.9	33.6	16.6
56	40	30.7	20.0	27.1	22.2

GC-FID: column DB-Wax (30 m x 0.25 mm x 0.15 μm), injection 0.01 μL, split 50:1, temperature gradient 40 °C (0 min) → 10 °C/min → 200 °C (12 min), carrier gas H_2 (1.2 mL/min). The compositions were obtained by comparing peak areas of both alcohols and acetates.

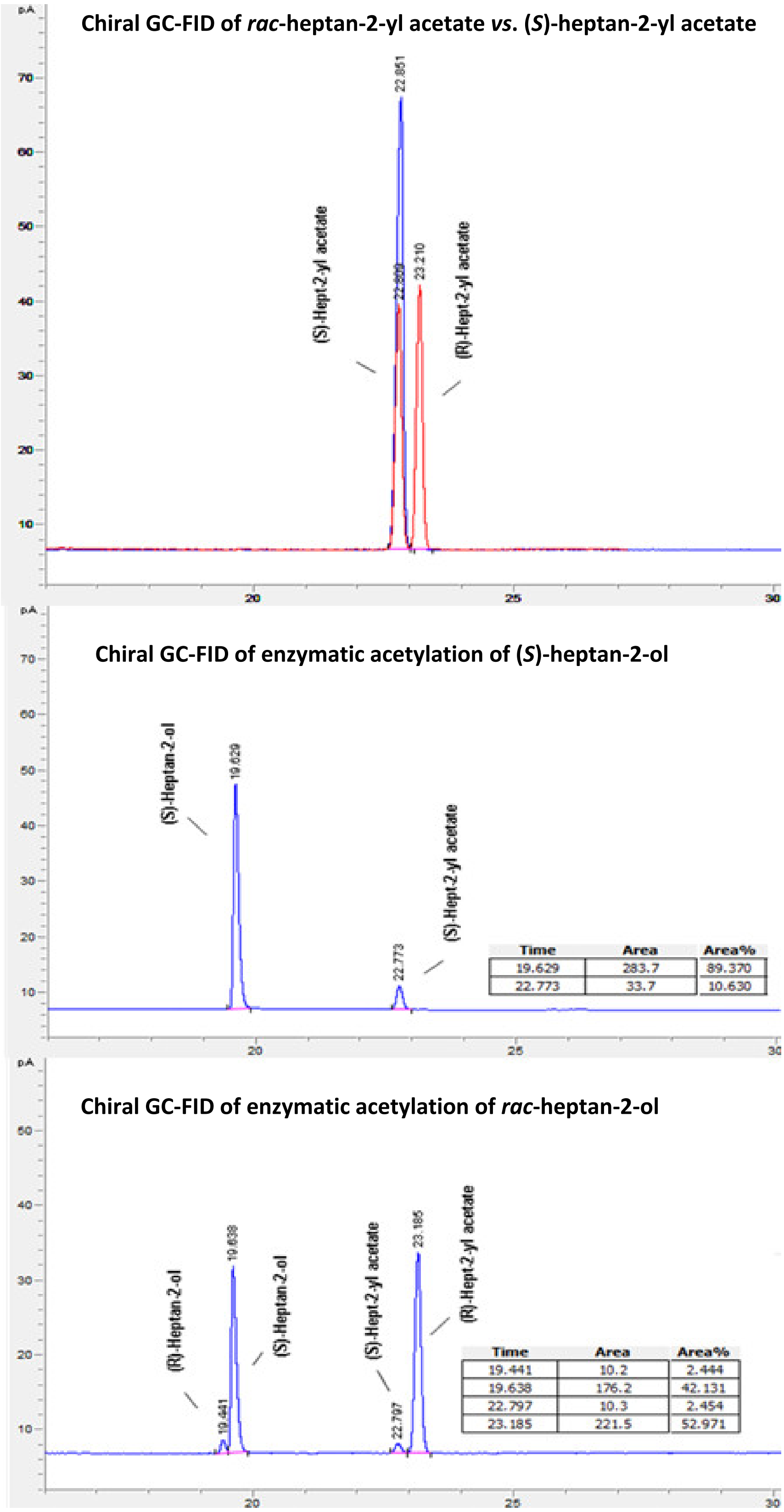

Figure S1. Chiral GC-FID analyses of heptan-2-ol (19) and heptan-2-yl acetate (20).

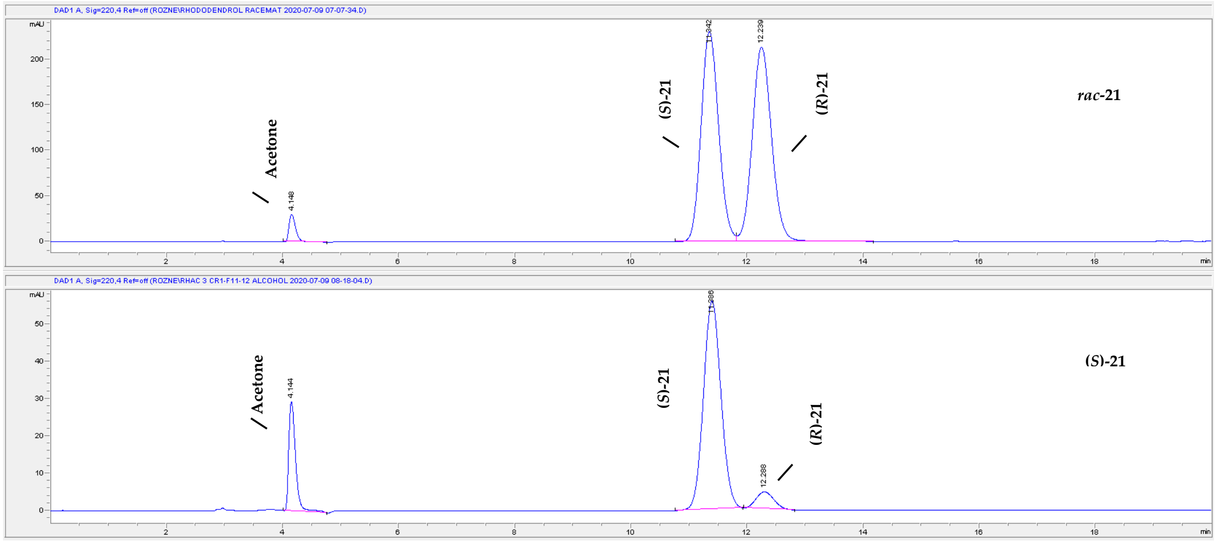

Figure S2. Chiral HPLC analyses of *rac*-rhododendrol *rac*-21 and enantioenriched (*S*)-rhododendrol (*S*)-21.

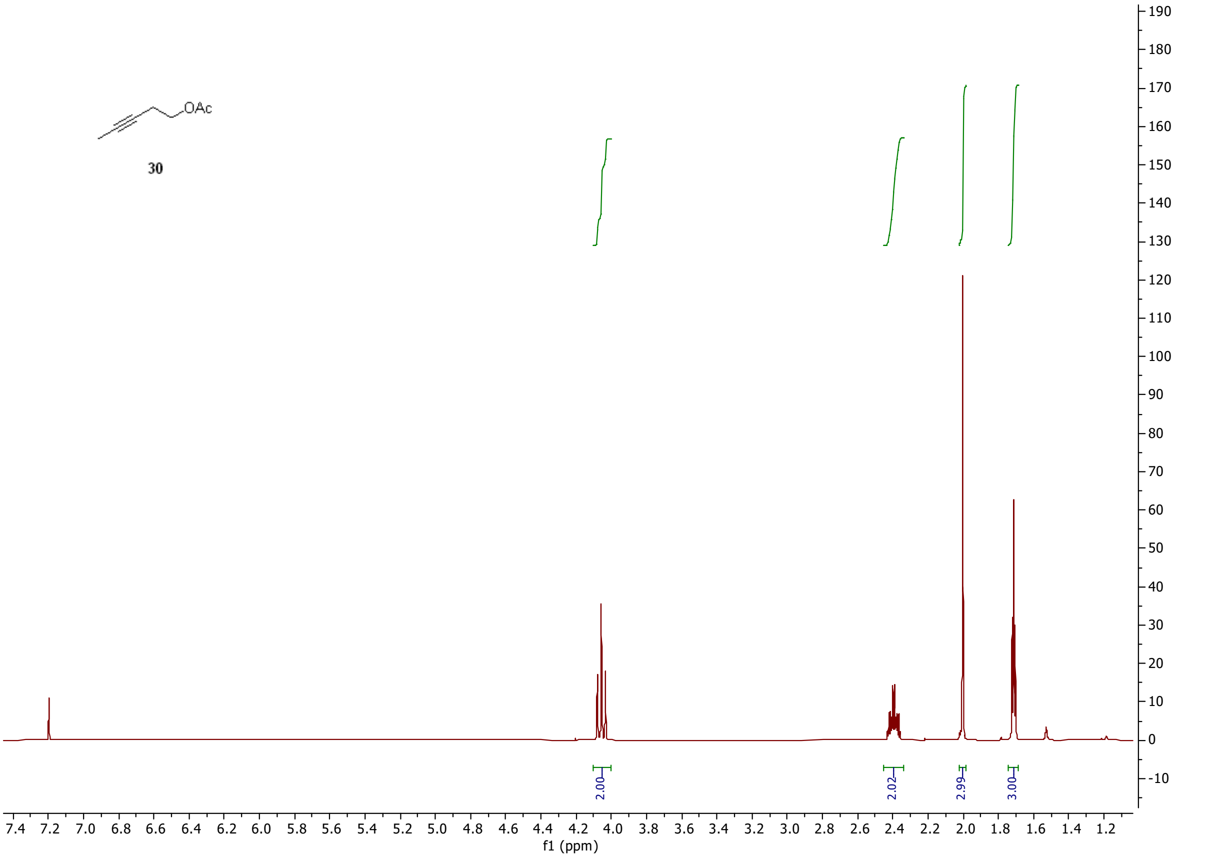

.

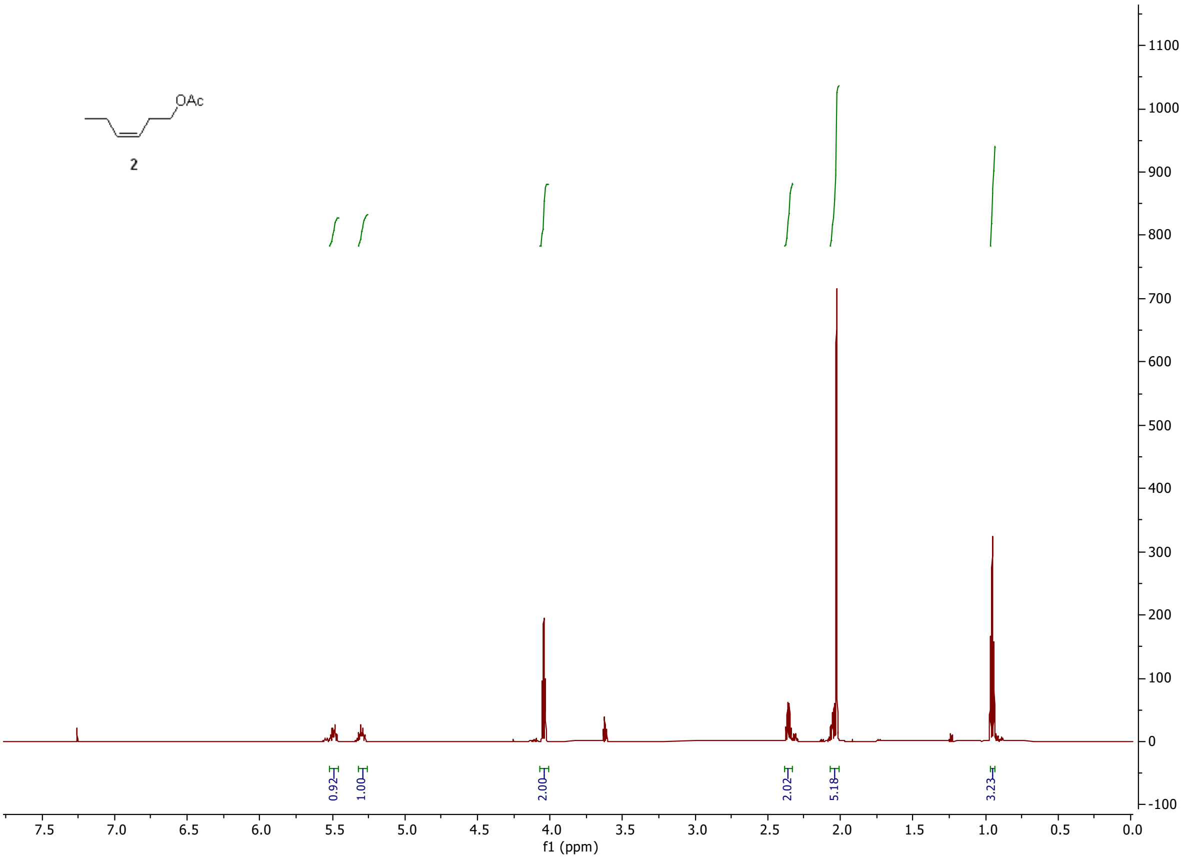
OAc
2
0.92
1.00
2.00
2.02
5.18
3.23
f1 (ppm)

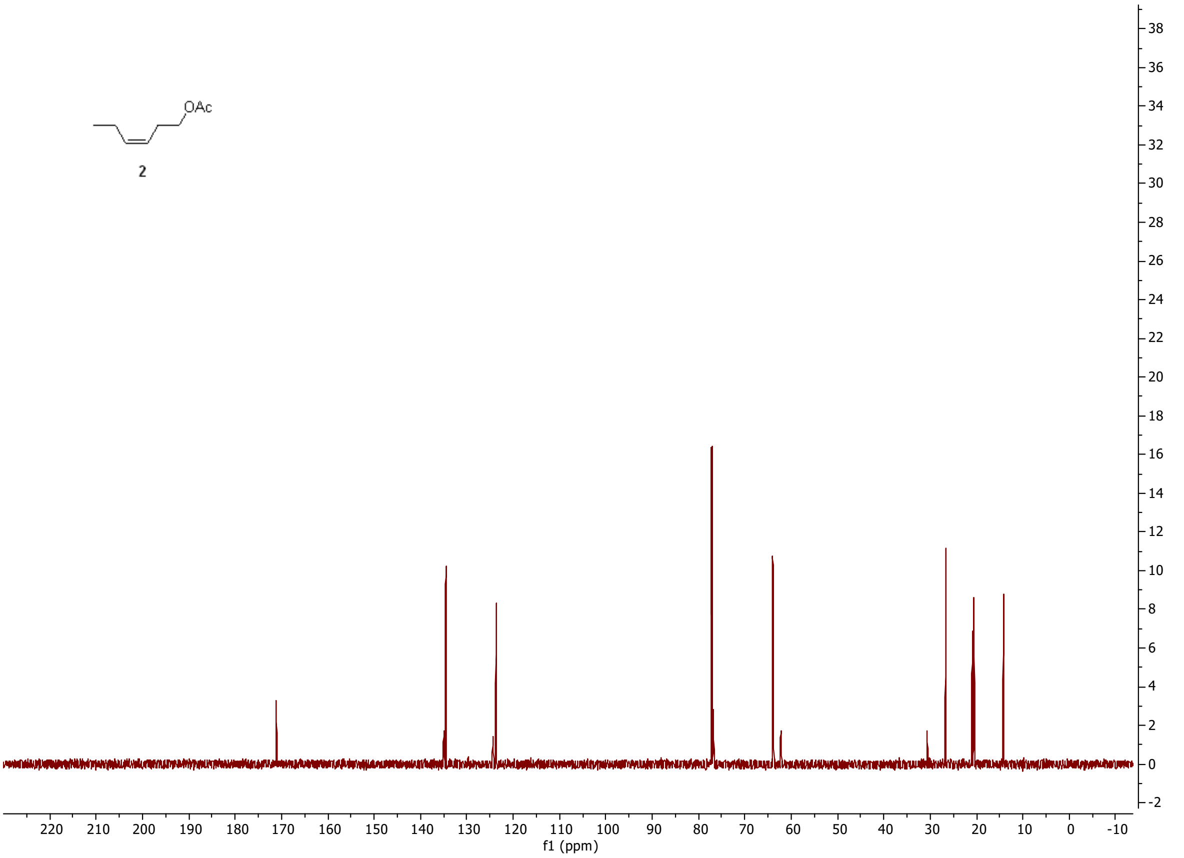
OAc
2
f1 (ppm)

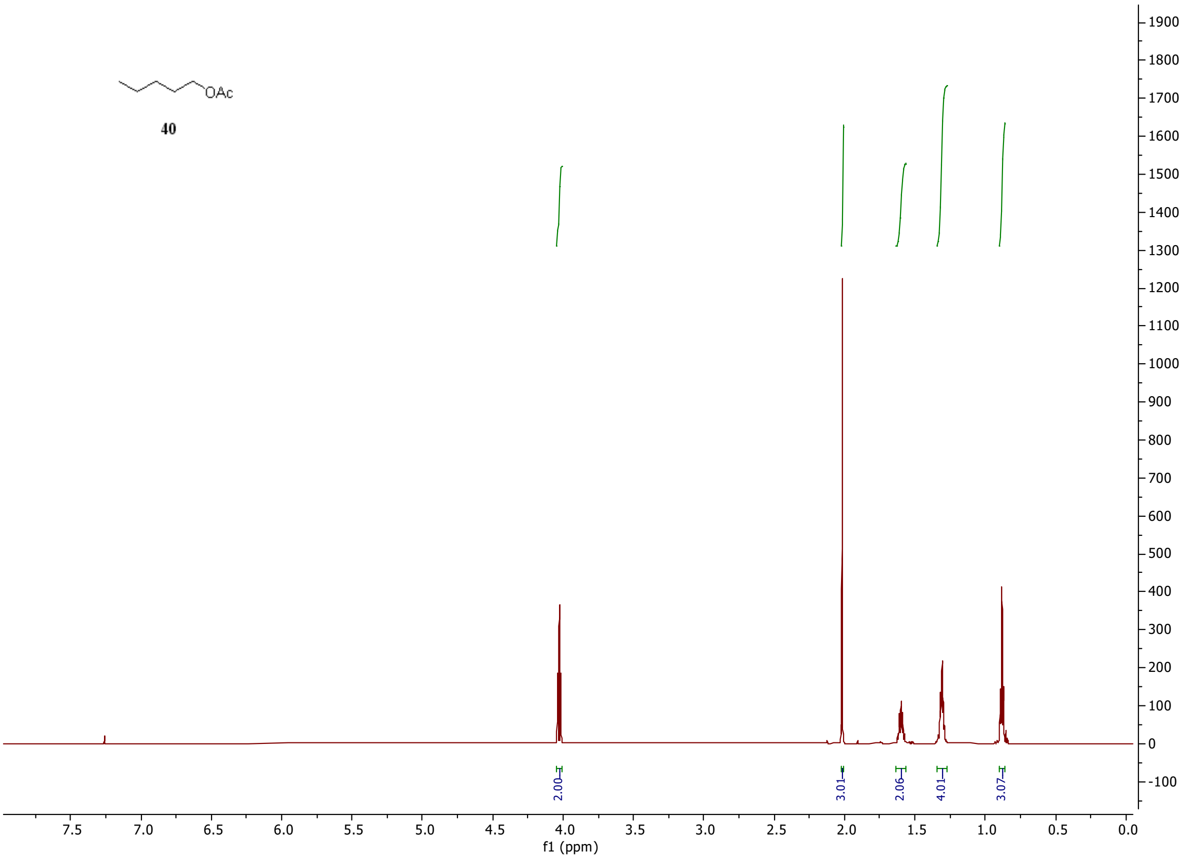

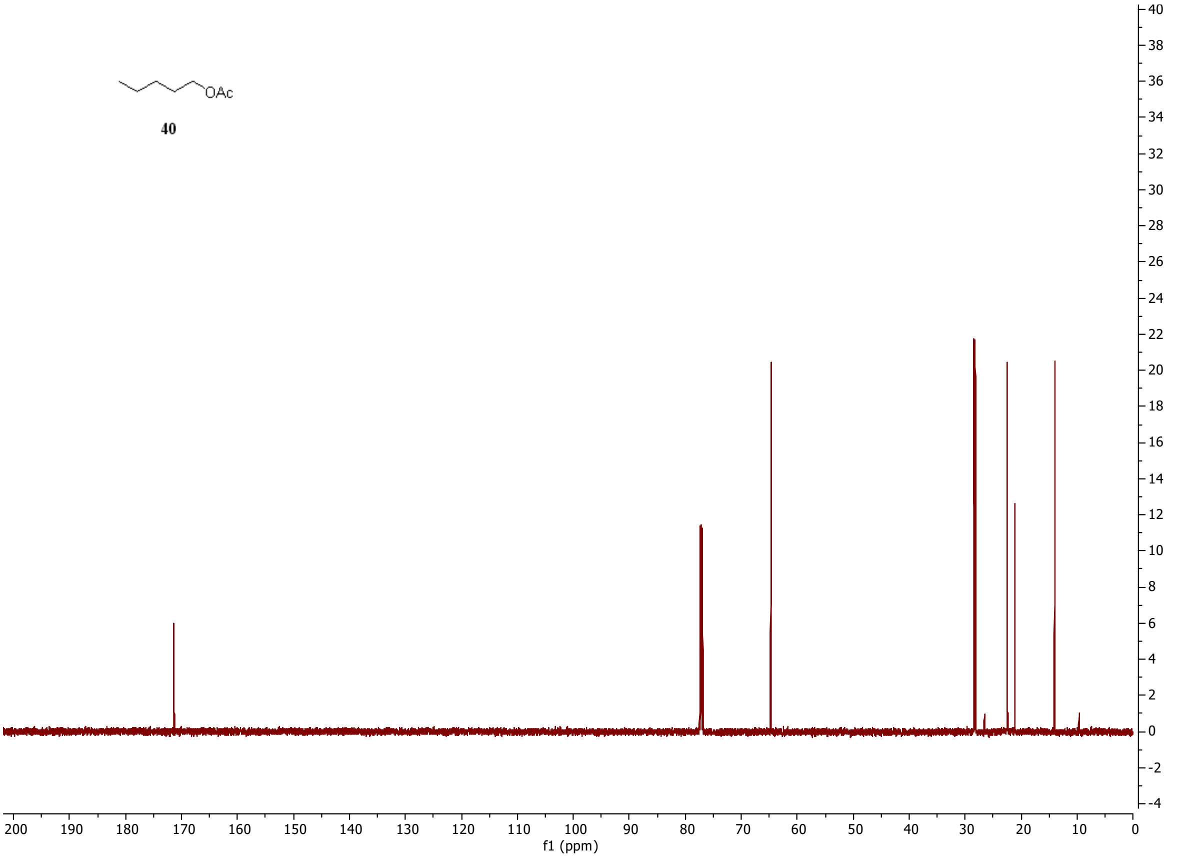

Figure S3. Copies of NMR spectra of isolated compounds.
